# Profiling of human IL-22+ T cell clones from patients affected with *Schistosoma mansoni*: Insights into macrophage regulation and liver fibrosis

**DOI:** 10.1371/journal.pntd.0013132

**Published:** 2025-05-30

**Authors:** Fernanda Scopelliti, Caterina Cattani, Roberto Gimmelli, Valentina Dimartino, Cristiana Lalli, Giuliana Papoff, Christian Napoli, Giovina Ruberti, Andrea Cavani

**Affiliations:** 1 National Institute for Health, Migration and Poverty INMP/NIHMP, Rome, Italy; 2 Institute of Biochemistry and Cell Biology, National Research Council (IBBC-CNR), Adriano Buzzati-Traverso Campus, Monterotondo, Rome, Italy; 3 National Institute for Infectious Diseases Lazzaro Spallanzani IRCCS, Rome, Italy; Xuzhou Medical University, CHINA

## Abstract

Tissue damage in *Schistosoma mansoni* infection results from a granulomatous, T cell–mediated response to parasite eggs, leading to liver fibrosis and portal hypertension. This immune response, initially Th1-dominated, progressively shifts toward a Th2 profile, contributing to hepatic stellate cell (HSC) activation and fibrosis. However, the precise regulatory mechanisms remain unclear. In this study, we analyzed T cell responses to soluble egg antigens (SEA) in 121 T cell clones (Tcc) from *S. mansoni*–infected patients. All clones produced high levels of IL-13 upon anti-CD3 stimulation; a minority secreted IFN-γ (n = 33) or IL-10 (n = 38). Notably, 51 clones co-produced IL-22 and IL-13. To investigate IL-22’s role, we examined IL-22 receptor (IL-22R) expression on human M0 and M2 macrophages. Both subsets expressed IL-22R, and its engagement triggered phosphorylation of p38, STAT3, and STAT5. IL-22 also downregulated IL-13–induced M2 markers (CD163, CD200R). Furthermore, IL-22 treatment of HSCs inhibited IL-13–driven collagen I/III production and cell proliferation. These results suggest that IL-22–producing T cells modulate Th2 macrophage polarization and directly suppress fibrogenesis in HSCs. IL-22 may thus act as a regulatory cytokine counteracting liver fibrosis during schistosomiasis.

## Author summary

Schistosomiasis poses a significant public health challenge, especially in tropical and subtropical areas, greatly impacting patients’ well-being. Our investigation focuses on the immunological mechanisms underlying liver fibrosis. We emphasize the vital role of cytokines like IL-22 in shaping the immune response. Although Th2 responses are critical for controlling the parasite, they can also result in considerable liver damage and complications, such as portal hypertension. Gaining a better understanding of how to modulate these immune responses could lead to new therapeutic strategies to prevent liver fibrosis, ultimately enhancing patients’ quality of life. The clinical relevance of our research is substantial. Currently, there are no effective treatments for liver fibrosis associated with schistosomiasis. Our findings could provide a scientific foundation for developing targeted treatment options, thereby improving disease management.

## Introduction

Schistosomiasis is a parasitic disease that affects more than 200 million people around the world (WHO) [1]. It is estimated that 5–10% of infected individuals develop severe forms of the disease, which are characterized predominantly by the granulomatous reaction and consequent fibrosis, in particular located in the liver and intestines in the case of Schistosoma mansoni and S.aponicum and in the urinary tract in the case of S. haematobium [2]. *S. mansoni* releases eggs that pass through the intestinal wall and are excreted in the feces. If deposited in fresh water, eggs may infect an appropriate species of snail, thus propagating the life cycle [3]. Occasionally, the large number of eggs produced by female schistosomes can become lodged in the intestinal wall or transported by the blood in the liver [1,4–6]. Once trapped into the liver vessel mature eggs recruit inflammatory cells and provoke the formation of a granuloma, that is an aggregate of different cells, especially neutrophils, lymphocytes, macrophages, eosinophils and fibroblast [4,7–10].

T lymphocytes, particularly CD4^+^ T cells, are essential component of the immune response against Schistosoma species and for the resistance to infection. Initially, the T cell response targets worm antigens, being dominated by Th1 lymphocytes. As female worms begin producing eggs, a strong type 2 T cell response focuses on egg antigens, characterized by the production of IL-4, IL-5, IL-9, and IL-13 [7,11]. Th2 cells are critical for orchestrating the immune response to Schistosoma and promoting antibody production against parasite. On the other side, their activity contributes to pathological conditions such as granuloma formation and fibrosis, highlighting the dual nature of Th2 responses in schistosomiasis. Indeed, cytokines produced by Th2 cells can promote collagen and other fibrosis-related genes in hepatic stellate cells (HSCs). Elevated levels of IL-13 in chronic schistosomiasis patients with liver fibrosis are associated with disease progression [12]. Conversely, in IL-13-deficient mice infected with *S. mansoni,* liver fibrogenesis is significantly diminished. Similarly, S. japonicum infection activates the IL-4 and IL-13 signaling pathway in the liver, influencing genes involved in extracellular matrix remodeling, such as Mmp2 and Mmp9. Mice lacking IL-4Rα, the receptor for IL-4 and IL-13, show reduced production of protective IgG1 and IgE antibodies and cytokines like IL-10 and IL-13, leading to increased susceptibility to *S. mansoni* infection [13,14].

Th2 cells are also pivotal for promoting macrophage differentiation that helps to control infection, but is strongly associated with granuloma formation [15]. Schistosoma granulomas are primarily composed of M2 macrophages [16]. Research by Zhu et al. [17] demonstrated that peritoneal macrophages from healthy mice expressed elevated levels of M2 chemokines (CCL2, CCL17, and CCL22), as well as IL-10 and Arg-1, when stimulated with *S. japonicum* soluble egg antigens (SEAs). M2 macrophages are polarized by the Th2 cytokines IL-4 and IL-13 mainly by activating STAT6 [18,19] and have a role in controlling pro-inflammatory pathology, while promoting the generation of fibrotic pathology. The presence of M2 macrophages can also influence Th2 responses, creating a feedback loop that sustains the Th2-dominated environment necessary for continued M2 polarization and control of the parasite [20].

Other T cell subsets contribute to the modulation of the immune response to Schistosoma. In particular, Tregs produce anti-inflammatory cytokines such as IL-10 and TGF-β, which help to suppress excessive immune responses and facilitate the transition from a Th1-dominated immune response to a Th2 response [21]. Moreover, Schistosome eggs promote the production of IL-22 transcripts while inhibiting the accumulation of IL-22-BP transcripts in schistosome-infected mice. Additionally, schistosome eggs selectively stimulate the production of IL-22 in blood leukocyte cultures from individuals chronically infected with *S. japonicum* [22].

Hepatic stellate cells (HSCs) play a critical role in periportal fibrosis following *S. mansoni* infection by remodeling the extracellular matrix and causing excessive collagen deposition. Activated HSCs are significant sources of extracellular matrix components and can secrete various pro-inflammatory and pro-fibrogenic cytokines. Th2 cytokines enhance collagen production and activate HSCs, further driving fibrosis development. The interaction between Th2 cells and HSCs creates a feedback loop that sustains the fibrotic environment [23–25].

Contrary to earlier reports, fibrosis can be effectively suppressed and potentially reversed. The regression of liver fibrosis is generally achieved through the inhibition of collagen production, which prevents the transformation of epithelial cells into myofibroblasts (MFBs). Additionally, activating immune cells to HSCs or promoting a shift in the immune response from a Th2 to a Th1 profile can further reduce inflammation and fibrosis. Moreover, promoting apoptosis or senescence in activated HSCs can decrease their numbers and activity, leading to regression of fibrosis [26].

The aim of the study was to assess the cell-mediated immune response to *S. mansoni* egg antigens and their roles in both promoting and mitigating fibrosis during schistosomiasis, potentially leading to insights for targeted therapies.

## Methods

### Ethics statement

Animal work was approved by the National Research Council, Institute of Biochemistry and Cell Biology Animal Welfare Committee (OPBA) and by the competent authorities of the Italian Ministry of Health, DGSAF, Rome (authorization nos. 336/2018-PR, 667/2023-PR). All experiments were conducted in respect to the 3R rules according to the ethical and safety rules and guidelines for the use of animals in biomedical research provided by the relevant Italian law and European Union Directive (Italian Legislative Decree 26/2014 and 2010/63/EU) and the International Guiding Principles for Biomedical Research Involving Animals (Council for the International Organizations of Medical Sciences, Geneva, Switzerland).

### Patients

Adult patients (n = 8) suffered from chronic Schistosomiasis of at least 6 months before sample collection. For each patient, venous blood was collected after written informed consent. Schistosomiasis was diagnosed using serology Western blot (WB IgG) containing a pool of antigen of worms of both species (SCHISTO II WB IgG, LDBIO, Lyon, France), and a rapid diagnostic test (RDT): Schistosoma ICT IgG-IgM (SCHISTOSOMA ICT IgG-IgM, LDBIO, France). Active infection was further confirmed by detecting circulating Schistosoma mansoni DNA by real-time PCR using QIAmp Circulating Nucleic Acid kit (Qiagen, Hilden, Germany) as previously described [27]. Specific primers were used (fw CCGACCAACCGTTCTATGA, rv CACGCTCTCGCAAATAATCTAAA). The analysis of PCR was performed with Roche LightCycler.

The study was approved by the ethical committee of the Ministry of Health (AOO-ISS 02/08/2019 0024068). Written informed consent was obtained from all subjects involved in the study.

### Life cycle *S. mansoni* maintenance and eggs preparation

A Puerto Rican strain of *S. mansoni* was maintained by cycling within albino Biomphalaria glabrata, as the intermediate host, and ICR (CD-1) outbred female mice as the definitive host, as previously described [28]. Mice were housed under controlled conditions at 22°C; 65% relative humidity; 12/12 hours light/dark cycle; standard food and water ad libitum. Female ICR (CD-1) outbred mice (Envigo, Italy) infected 7–8 weeks previously with 200 double sex cercariae were euthanized with intraperitoneal injections of Tiletamine/Zolazepam (800 mg/kg) + Xylazine (100 mg/kg) and adult parasites were harvested by reversed perfusion of the hepatic portal system and mesenteric veins. Enriched mature eggs were isolated from livers of heavily infected mice, perfused of parasites, as previously described [29].

### SEA preparation

The protocol adopted for SEA preparation has been previously described [29]. Briefly, *S. mansoni* eggs were mixed with an appropriate volume of sterile PBS (100,000 eggs/ml.). Eggs were homogenate on ice using the pre-chilled homogenizer, and then the mixture was centrifuged at 4 °C and 15000 × g for 30 min. Supernatant was ultracentrifuged for 90 minutes at 100,000 x g at 4°C. Finally, the supernatant was sterilized with a 0.22 μm filter, and the protein concentration of SEA was determined with a Bradford Protein Assay Kit (Bio-Rad, Hercules, USA)

### T cell culture and proliferation

PBMC were obtained upon Lympholyte-H (Cedarlane, Canada) gradient from 10 ml of blood sample

and then cultured in presence of 10μg/ml of SEA antigen for 5 days in RPMI 1640 supplemented with 2 mM glutamine, 1% penicillin/streptomycin (Corning,USA), and 5% human serum (Sigma-Aldrich, RPMI complete) and subsequently labeled with click it proliferation assay (Thermofisher, USA). (EDU a nucleoside analog of thymidine) was added to the culture media at 10 nM for 2 hours. After labeling with PBS/BSA, cells were fixed and marked with FITC antibody following the instructions of the company. Acquisition was performed using an Attune Nxt (Life-Technologies, California) cytofluorimeter.

### Isolation and expansion of T cell clones

T cell lines proliferating to SEA antigens were cultured in the presence of 30 U/ml IL-2 (proleukin Clinigen,Ireland) for additional 4–5 days and finally cloned by limiting dilution (0.6 cells/well in 96-well U-bottom microtiter plates) in RPMI complete 5% human serum and 10% heat-inactivated FBS (Hyclone, USA) on a feeder layer of mitomicinated PBMCs, 50 IU/ml IL-2, and 1% PHA-M (Gibco,Thermofisher Scientific, US). Fresh medium containing IL-2 was added 3 times per week, and T clones were restimulated with mitomicinated PBMCs and 1%PHA-M every 3 weeks.

### Detection of cytokine release by T cell lines and T cell clones

Supernatants from T cell lines activated with 10 μg/ml SEA were collected at day 5. Supernatants from T cell clones activated with T cell Transact (Miltenyi Biotec, Germany) were collected at 48 h. Supernatant contents of IFN-γ, IL-4, IL-13, IL-10, IL-17, IL-22, IL-33 were measured using commercially available sandwich ELISA kits (all from R&D systems, Minneapolis).

### Macrophage culture and phenotype characterization by cytofluorimetry

PBMC were obtained after gradient centrifugation upon Lympholyte-H from buffy coats (Transfusional Unit, S. Filippo Neri hospital, Rome). Monocytes were positively selected by incubation with immunomagnetic beads coated with anti-CD14 (Miltenyi Biotec, Germany), according to the manufacturer’s protocol, and cultured in RPMI 1640 supplemented with 200 mM glutamine,1% penicillin/streptomycin (all from Corning,USA), 10% of fetal bovine serum (Sigma-Aldrich, USA) (complete medium) and 10 ng/mL colony stimulating factor 1 (CSF-1) for 6 days (PeproTech,UK) to obtain M0 macrophages. M2 polarization was achieved by further treating M0 macrophages with 20ng/ml IL4 (PeproTech) for 48 hours.

Macrophages M0 were cultured for 48 hours in complete medium in the presence or in the absence of 20ng/ml IL-13 alone or in combination with 25ng/ml IL-22 (Peprotech, USA). For surface marker staining, cell populations were washed with PBS and stained with FITC-, PE-, PerCP-conjugated mAb for 20minutes. Staining with matched isotype control Ig was used as control. Acquisition was performed using an Attune Nxt (Life-Technologies, California) cytofluorimeter. Analysis was performed using Flow logic software (Miltenyi) according to Guidelines for the use of ﬂow cytometry and cell sorting in immunological studies [30]. mAb anti-human CD-200RFITC (OX-110), CD-206 PE (FN50), CD-163 APC (B27 and R4-6A2), HLA-DR FITC (LN3), were purchased from BD Biosciences, California; IL-22-R APC(# 305405); CD-14 PE (# 134620) was purchased from RD; mouse IgG isotype controls were purchased from BD Biosciences.

### Western blot analysis

Macrophages were cultured as described above and treated at different times with 25ng/ml IL-22; total protein extracts were obtained at different time points using RIPA lysis buffer, accordingly to standard procedures, as previously reported [31]. 20 μg Proteins from total lysates were resolved by SDS-polyacrylamide gel electrophoresis, transferred to nitrocellulose membrane, blocked in 5% bovine serum albumin, and blotted with the following antibodies: anti-phospho ERK1/2 (Thr202/Tyr202) (CST,USA), anti-phospho P38 alpha (Thr 180,/Tyr 182) (Invitrogen,USA), anti-phospho Stat3 (Tyr705) (CST,USA), anti-phospho Stat5 (Tyr694) (CST,USA), GAPDH (Santa Cruz Biotechnology, USA).

As secondary antibodies, anti-mouse, anti-goat, or anti-rabbit Ig Abs conjugated to horseradish peroxidase were used and detected by the ECL-plus detection system (Thermofisher Scientific), by using ChemiDoc MP Imaging System (Bio-Rad, Hercules, CA, USA). Densitometric analyses were performed using Image J software.

### Cytokine effect on fibrogenic activity of hepatic stellate cells

Immortalized Hepatic stellate cells (HSC) (Innoprot, Spain) were maintained in stellate cell medium complete (Innoprot).

HSC were treated with IL-13 (20 ng/ml) and IL-22 (25 ng/ml) alone or in combination for 24 hours.

20 μg Proteins from total lysates were resolved by SDS-polyacrylamide gel electrophoresis, transferred to nitrocellulose membrane, blocked in 5% non-fat dry milk, and blotted with the following antibodies: Collagen I α 1, Collagen III α1,(all from Elabscience, USA), Actin (Santa Cruz Biotechnology, USA).

As secondary antibodies, anti-mouse, anti-goat, or anti-rabbit Ig Abs conjugated to horseradish peroxidase were used as described above and detected by the ECL-plus detection system (Thermofisher Scientific) by using ChemiDoc MP Imaging System (Bio-Rad, Hercules, CA, USA). Densitometric analyses were performed using Image J software.

## Results

### Schistosoma patients

Eight patients afferent to the INMP outpatient clinic with positive circulating IgG towards Schistosoma antigen (western blot) were included in the study. To confirm the active infection of *S. Mansoni*, molecular test based on the detection of *Schistosoma* cell free DNA in the serum was performed, as previously described [27]. Blood samples of African healthy donors were used as a control.

### PBMC from Schistosoma positive patients are activated by SEA antigen

**Fig 1:**
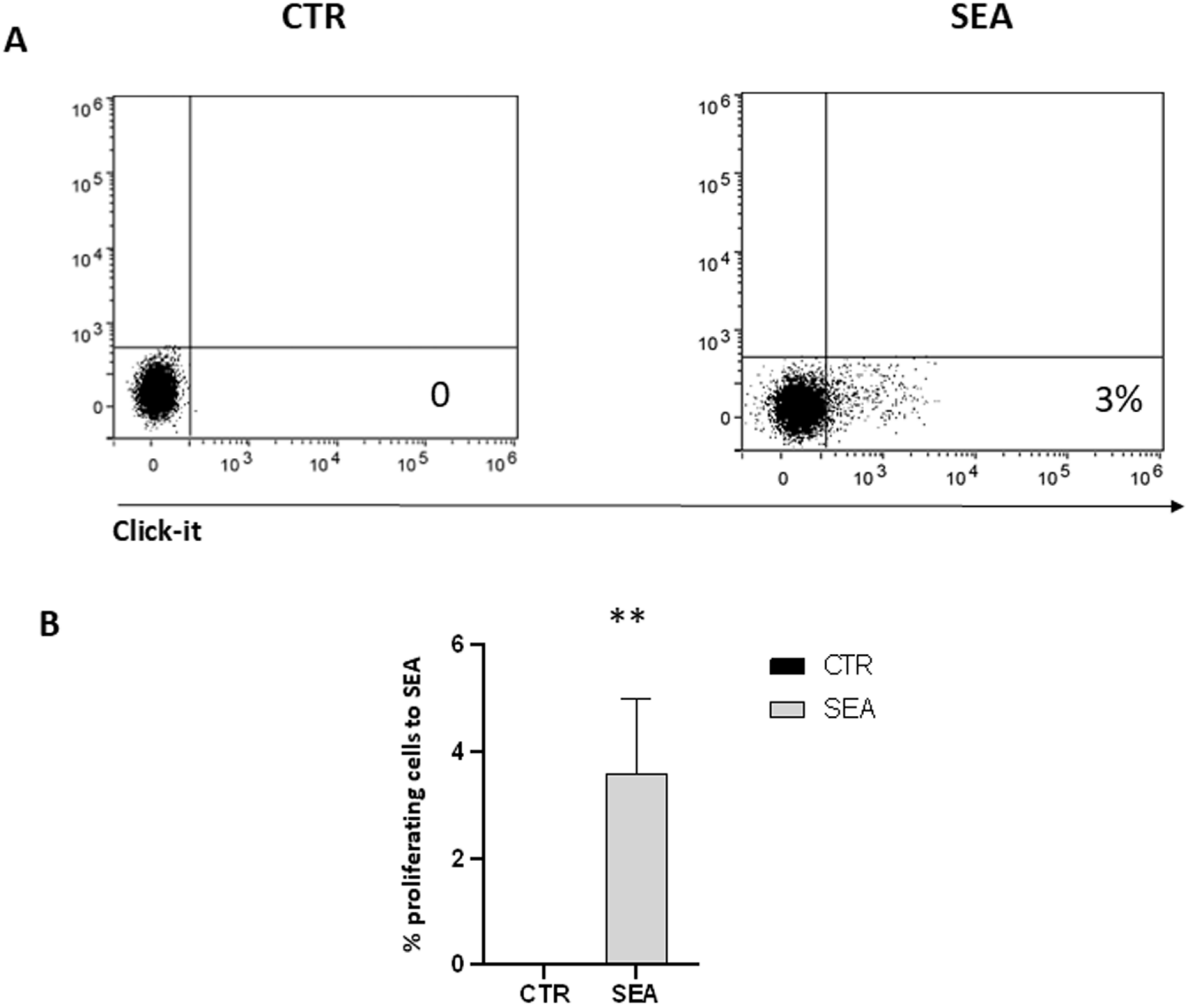
Cells obtained from 8 different donors were collected, treated with SEA (10 μg/ml) for 5 days and proliferation analyzed by FACS, as described in Methods. The plots show the data obtained in the presence (SEA) and absence of antigen (CTR) in one representative experiment out of 8. In B the percentage of proliferating cells from each donor compared to non affected control (*p ≤ 0.05 as calculated by paired Student’s t test) is shown.

**Fig 2:**
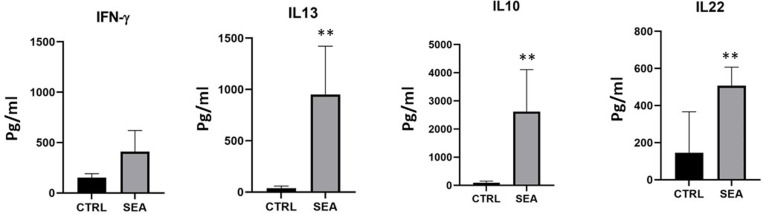
PBMC from 8 patients were co-cultured with or without SEA (10 μg/ml) and culture supernatants were collected at day 5. Cytokine content was measured by ELISA Data are expressed as mean pgs/100.000 cells ± SD of triplicates (*p ≤ 0.05 as calculated by paired Student’s t test).

To investigate whether T cells isolated from affected patients specifically respond to eggs antigens, PBMC from 8 patients were incubated in the presence or absence of 10 μg/ml of SEA antigen for 5 days, and proliferation assessed by cytofluorimeter using the click it test. Percentage of responding cells in the presence of SEA antigen varied between 2.35 and 5.79 (average of 2.49 ± 0.6%; -; proliferation of T cell from non-affected control was 0 (Fig 1). To investigate the cytokine profile of PBMC, supernatants of cell cultures were collected and analyzed by ELISA. Results shown obvious differences between controls and SEA treated samples: in particular, in the presence of SEA antigen, cells can secrete large amounts of IL-10, IL-13, as already reported and, unexpectedly, also high amounts of IL-22 cytokine (Fig 2); In contrast, no detectable amount of IL-17, IL-4 and IL-33 was detected.

### Characterization of T cell clones expanded upon SEA antigen activation in Schistosoma affected patients

**Fig 3:**
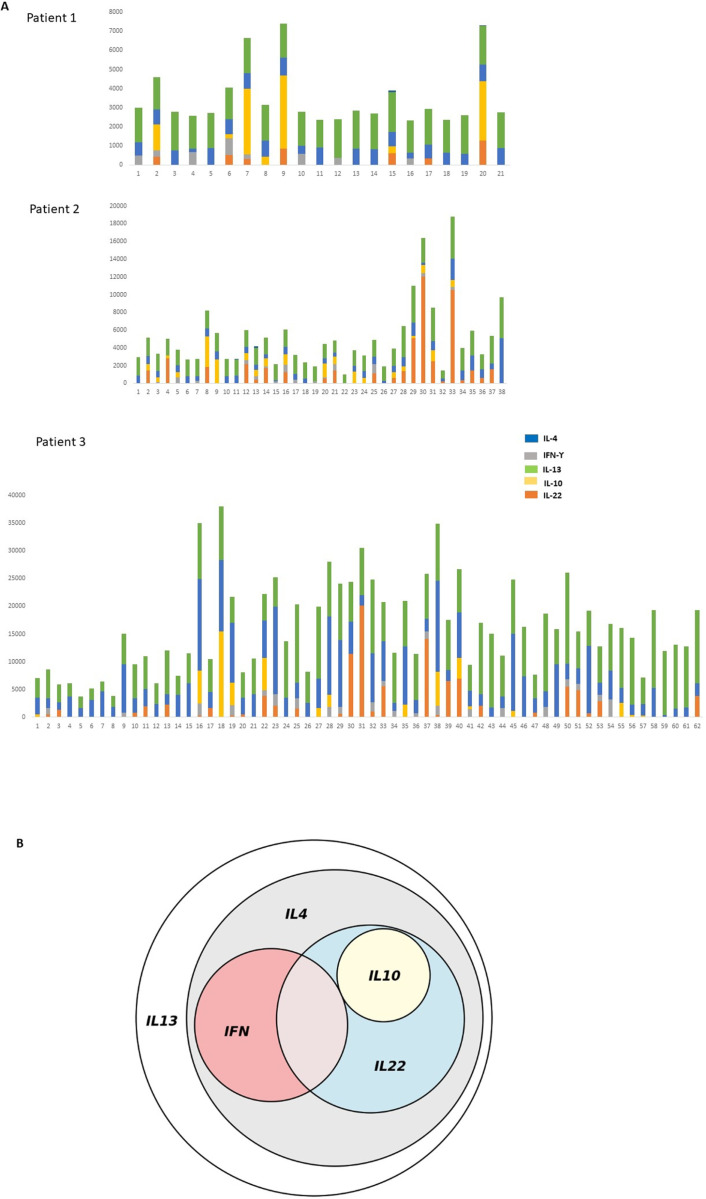
A) T cell lines obtained from PBMC of Schistosoma patients treated with SEA (10 μg/ml), cloned by limiting dilution and analyzed by ELISA after 48h incubation with anti-CD3 Ab. Bars of the graph represent the number of pg/ml of IL-22, IFN, IL-10, IL4,IL-13,IL17 secreted by each clone obtained from 3 different donors. B) Euler diagram of cytokines expression of T cell clones obtained from 3 different donors.

PBMC cultured 5 days in the presence of SEA antigen were further cultured for 5 days in the presence of 30U/ml IL-2 to expand CD25 + preactivated T cells and finally cloned by limiting dilution as described in material and methods. A total 121 T cell clones from 3 different donors were obtained, and cytokine profile investigated by ELISA upon a 48 h activation in the presence of T cell Transact. As expected, all clones produced IL-13 whereas IL4 vas released by 117 clones. 45 clones produced in addition IFN-Υ and 43 produced IL-10. Surprisingly, 56 clones produced IL-22: among them 26 produced IL-13, IL-22 and IL-10 simultaneously, whereas 29 Tcc co-released IL-13 and IL-22 without IL-10 (Fig 3). No T clones produced IL-33.

### Macrophage express functional IL-22 receptor

**Fig 4:**
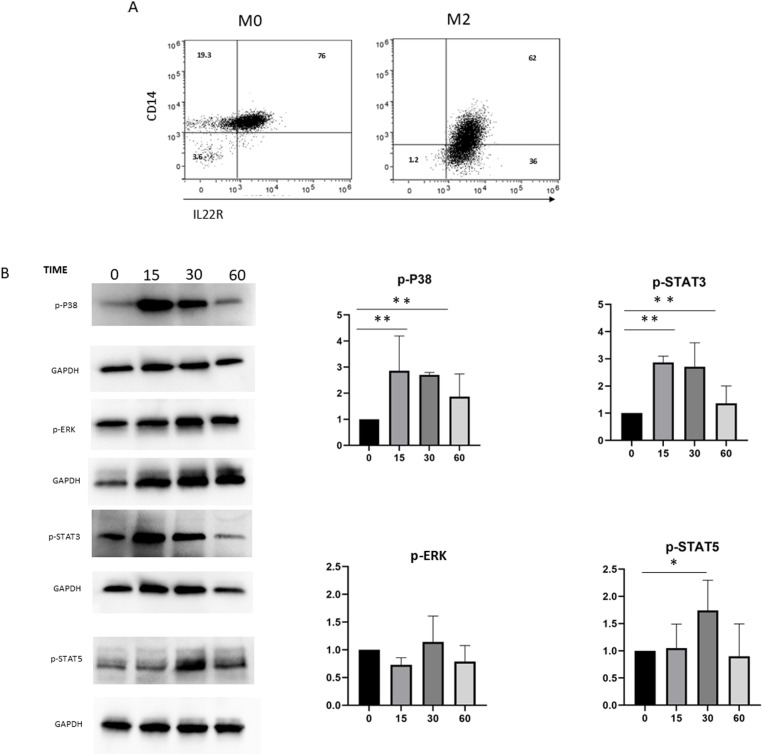
M0 and M2 macrophages obtained from 3 healthy different donors were analyzed by Flow cytometry, as described in methods. Plot show the expression of IL-22 receptor and CD14 in M0 and M2 macrophages. WB analysis was performed on protein lysates from M0 treated with 25ng/ml of IL-22 at different time point. The following antibodies were used: anti-phospho ERK1/2 (Thr202/Tyr202) cell signaling, anti-phospho P38 alpha (Thr 180,/Tyr 182), anti-phospho Stat3 (Tyr705), anti-phospho Stat5 (Tyr694), Gapdh was used as loading control. Numbers in bold represent fold increase of protein expression vs control. Figure represent one representative experiment of 3. Graphs represent the mean values of densitometric intensity (D.I.) of each band ± SD of DCs normalized to GAPDH(*p ≤ 0.05 **p ≤ 0.005, as calculated by paired Student’s t test).

Macrophages are the main cellular component of the granulomatous response to the eggs and they play a key role in development of hepatic fibrosis. To determine whether IL-22 could affect macrophage function in the context of liver granuloma, expression of IL-22R1 on M0 and M2 macrophages subsets was evaluated by flow cytometry and western blot. As illustrated in Fig 4 A, macrophages activated with CSF-1 express IL-22 receptor, both in cytofluorimetry and western blot although the % of expression was donor dependent. Importantly, the expression of the receptor was confirmed also in M2 macrophages (Fig 4). To examine whether the presence of receptor are reflected in the functional assays, macrophages M0 were treated at different time points with IL-22 (20 μg/ml) and western blot analysis of phosphorylated proteins were performed. As reported in Fig 4B, IL-22 induced upregulation of phospo-STAT3, phospo-STAT5, phospo-p38 at different time points, while p-erk was not modulated (Fig 4B).

### IL-22-induced schistosoma decreases M2 response

**Fig 5:**
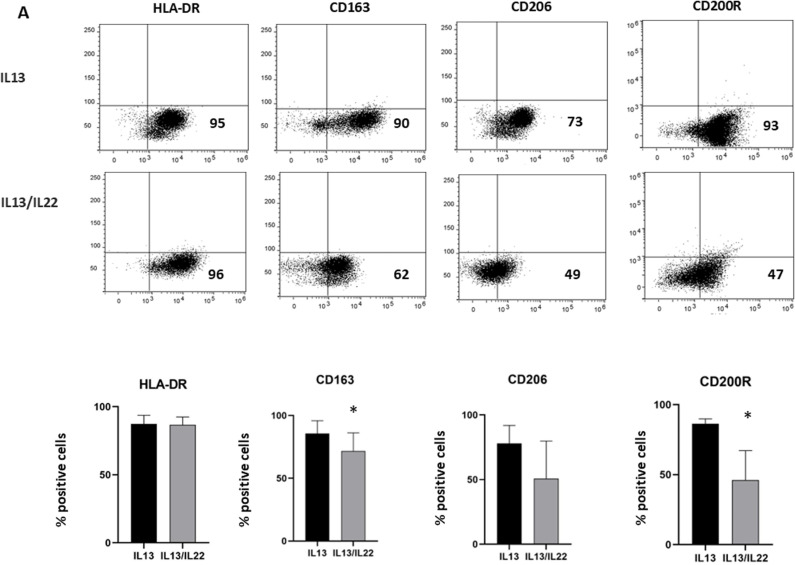
A) M0 macrophages obtained from 3 different healthy donors were cultured 48h in the presence of IL-13 (20 ng/ml) with or without IL-22 (25 ng/ml) and analyzed by FACS, as described in Methods. The plots show the data of one representative experiment out of 3. B) Graphs show the percentage of cells expressing the indicated markers at 48h (*P < 0.05).

Macrophages have been shown to be a key component for granuloma formation; in particular, M2 macrophages play critical roles in the maintenance of granuloma and subsequent fibrosis. IL-13 is a key regulatory cytokine for Th2 cell-mediated granuloma induced by *S. mansoni* eggs, while is unknown the role of IL-22. To investigate whether IL-22 could influence the polarization of macrophages in a context of fibrosis induced by Schistosoma, primary human macrophages were treated with IL-13 in the presence or absence of IL-22, and expression of M2 related markers were investigated by cytofluorimetry. As shown in Fig 5, exposure to IL-22 in combination with IL-13 significantly decreased the expression of the M2 marker CD-163, CD-2OOR and a non-significant reduction of CD-206 were observed, while HLA-DR was not modulated.

### IL-22 modulate the profibrotic activity of IL-13 on HSC

**Fig 6:**
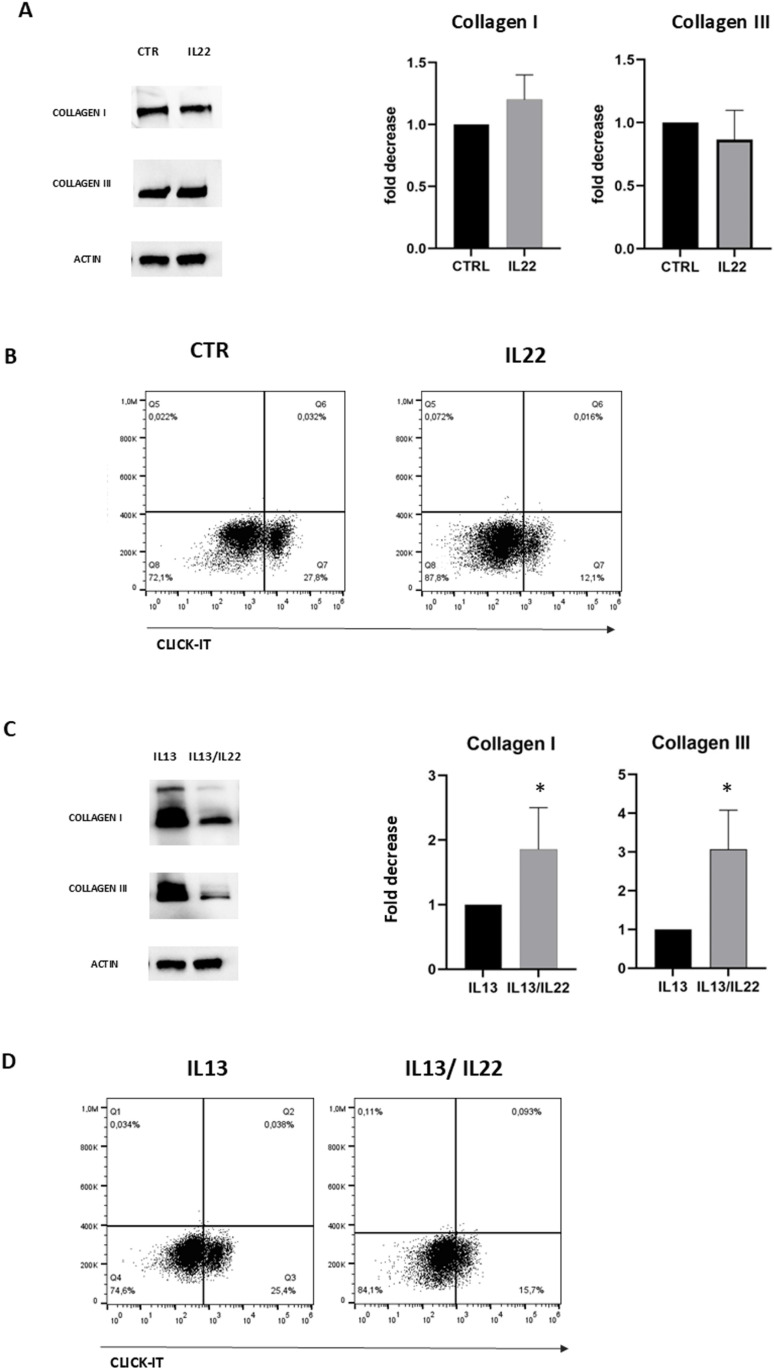
A) HSC were cultured for 24h presence or absence of IL-22 (25 ng/ml) and protein lysates were analyzed for Collagen I and III protein expression by western blots. Graph show the fold decrease of signals of a representative experiment out of 3. B) HSC were collected, treated with the cytokines, as described above, for 48h and cell proliferation was analyzed by FACS, as described in Methods. The plots show the data obtained in a representative experiment out of 3. C) HSC were cultured for 24h with IL-13 (20 ng/ml) in presence or absence of IL-22 (25 ng/ml) and protein lysates were analyzed for Collagen I and III protein expression by western blots. Graph show the fold decrease of signals of a representative experiment out of 3. D) HSC were collected, treated with the cytokines, as described above, for 48h and cell proliferation was analyzed by FACS, as described in Methods. The plots show the data obtained in a representative experiment out of 3.

In a preliminary series of experiments, we sought to assess the impact of IL-22 on hepatic stellate cells (HSC), which play a key role in fibrosis and tissue remodeling. The primary focus was to evaluate IL-22’s influence on collagen production and cell proliferation, two fundamental processes associated with the pathophysiology of fibrosis. Our Western blot analysis indicates that IL-22 has a negligible or highly subtle effect on collagen synthesis in HSC after 24h (Fig 6A). On the other hand, a marked reduction in cell proliferation was observed in flow cytometry following IL-22 treatment, suggesting a potential role for IL-22 in inhibiting the expansion of stellate cells (Fig 6B). HSC, when influenced by type 2 cytokines, can over-activate the fibrotic tissue pathway. To investigate whether IL-22 could modulate the function of fibroblasts in a Th2 context, HSC were treated with IL-22 in combination with IL-13, and collagen production was then assessed by WB. Results show that at 24 hours, IL-22 decreased the production of both type I collagen (4.1 ± 0.7-fold increase) and type III collagen (2.1 ± 1.1-fold increase) by HSC compared to controls, indicating a protective role for IL-22 in regulating the production of extracellular pro-fibrotic molecules; a marked reduction in cell proliferation was observed in flow cytometry was confermed(Fig 6C-D).

## Discussion

To better understand the immune mechanisms involved in the pathogenesis of schistosomiasis, we investigated the T cell response to SEA in patients affected by *S. mansoni* infection, aiming to clarify how the immune system could contributes to liver fibrosis. Our results confirmed the predominant Th2 response to egg antigens. Interestingly, a significant number of T cell clones isolated from patients released IL-22, which modulates macrophage M2 polarization, collagen production and proliferation of hepatic stellate cells.The ability of parasite, like schistosomiasis, to orchestrate the immune system of their host is crucial for their survival, enabling them to evade excessive immune reactions [32]. Previous studies, primarily conducted in mouse models, have established a strong T-helper 1 (Th1) immune response directed against the worms in the early acute phases of infection [33]. This phase is characterized by pro-inflammatory cytokines, including tumor TNF-α, IFN-γ, IL-1, and IL-6. Approximately 5–6 weeks post-infection, schistosomula develop into mature worms, and egg deposition begins. While most eggs are excreted in feces, some reach the liver, where they become trapped in hepatic sinusoids, stimulating a reactive inflammatory response. In this chronic phase, type 1 cytokines gradually decrease, while Th2-type cytokines IL-4, IL-5, and IL-13 increase, primarily directed against egg antigens SEA [34]. SEA is a complex mixture of proteins, glycoproteins, and glycolipids, whose composition may vary depending on the egg stage. *In vitro* studies have demonstrated that SEA components interacting with local dendritic cells are directly responsible for the Th1-Th2 skewing [35,36].

Murine studies have shown that the transition from a pro-inflammatory Th1 to a Th2 response is protective for the host [37]. Indeed, in mice deficient in IL-4 or in a Tamoxifen-induced IL-4 receptor α (IL4Rα)-deficient model, the persistence of a strong Th1 response leads to severe disease and high mortality [38]. Although a Th2-dominated immune response is generally beneficial for the elimination of parasites, in the case of schistosomes, the protective effect of a Th2 immune response does not lead to parasite clearance, making the situation less clear. Indeed, Th2 cytokines, particularly IL-13, are primarily responsible for the fibrotic process following the deposition of schistosome eggs in the liver, being this fibrotic process detrimental to the host, leading to hepatic and periportal fibrosis, which can obstruct blood flow and result in portal hypertension and potentially fatal esophageal bleeding [39]. This indicates that the Th2 response is a double-edged sword: while it aids in eliminating or containing the parasite and reducing excessive inflammation, it can also lead to organ damage.The activation of hepatic stellate cells (HSCs), which constitute about 5–8% of all liver cells, is a key factor in liver fibrosis. When stimulated by eggs, HSCs regulate the expression of fibrotic components such as collagen type I, collagen type III, and fibronectin, ultimately contributing to liver fibrosis. IL-13 promotes liver fibrosis through two main mechanisms: I) IL-13 produced by Th2 clones polarizes macrophages toward the M2 phenotype. These M2 macrophages contribute to fibrosis development, granuloma maintenance, tissue repair, and host survival. II) IL-13 acts directly on HSCs, promoting their production of extracellular matrix components. In murine models, inhibiting IL-13 has been effective in treating ongoing *S. mansoni* infection-induced fibrosis.

Thus, the Th2 response must be strictly regulated during schistosome infection to minimize adverse effects.

Our data demonstrate that peripheral blood mononuclear cells (PBMC) from chronically infected patients are activated in vitro by SEA antigens. T cell clones obtained through limiting dilution from SEA-stimulated cell lines are enriched in Th2 cells, characterized by substantial release of IL-13 and IL-4, with limited production of IFN-γ. Notably, a large number of these Th2 clones coproduce IL-22, either alone or in combination with IL-10, and in the absence of IL-17. IL-22 is a key signaling molecule involved in various physiological processes, from innate immune responses to tissue regeneration. The up-regulation and down-regulation of IL-22 have significant consequences, defining its biological and pathological activities [40]. For instance, IL-22 protects epithelial tissues from damage and mediates tissue repair [41]; it also stimulates the production of antimicrobial peptides that limit microbial invasion through epithelial lesions [42]. Additionally, IL-22 has been reported to protect against liver injury in several chronic diseases. The IL-22 receptor (IL-22R) is expressed in various cell types, which underscores its multifaceted role in immune regulation and tissue homeostasis. IL-22R is prominently expressed by tissue resident cells, such as epithelial cells and fibroblasts [43,44]. HSCs also express abundant IL-22R. Notably, some subsets of T cells in specific conditionshave been shown to express IL-22R, indicating their potential responsiveness to IL-22 [45].

The expression of the IL-22 receptor on macrophages is not clear. Treerat and collegues have shown that during Mycobacteria tuberculosis infection human as well murine macrophages express the IL-22R [46]. In line with this observation, we found that in vitro polarized macrophage M0 and M2, being the latter the principal macrophage subpopulation in the shistosoma granuloma, do express functional IL-22R. IL-22R ligation by IL-22 transiently induces p38 and STAT3 phosphorilation. It is well-established that signaling cascades like those mediated by STAT3, STAT5, and p38 are often characterized by rapid, but transient, activation. Initially, IL-22 engagement leads to the activation and phosphorylation of these proteins, but as the signaling pathways progress, negative feedback mechanisms and phosphatase activity may lead to their dephosphorylation and downregulation by later time points [47].

Moreover, IL22 negatively modulates M2 polarization induced by IL-13 treatment by reducing the expression of CD163, CD200R, and CD206, while HLA-DR expression remains unaffected. Importantly, IL-22 controls excessive M2 polarization linked to the fibrotic process without inducing an M1 polarization, which could be detrimental.

Hepatic stellate cells are intimately associated to liver fibrosis. IL-13 promotes the transition of hepatic stellate cells from a quiescent to an active one, stimulating their proliferation and increasing their synthesis of collagen types I and III, which are key components of the extracellular matrix and responsible for the accumulation of fibrotic tissue that disrupt normal liver architecture. Our results demonstrate that IL-22 directly inhibits the production of collagen I and III and the proliferation of HSC induced by IL-13 [48].

Thus, overall, IL-22 exerts regulatory functions and could attenuate the consequences of the Th2 response in the chronic phase of Schistosoma infection by reducing macrophage M2 polarization and the pro-fibrotic activity of HSC. During the co-evolution of host and parasite, anti-inflammatory mechanisms have emerged, allowing the worm to suppress host immunity while ensuring host viability (thereby preserving its habitat), and enabling the host to eliminate the parasite without incurring “collateral damage.” Understanding the role of IL-22 is critical, as effective immunotherapies for schistosomiasis will necessitate a comprehensive understanding of the mechanisms underlying T cell-mediated liver immunopathology.

Currently, no effective drug exists for the treatment of schistosomiasis-related liver fibrosis, complicating our understanding of the pathogenesis and management of the disease. A Th1 phenotype could be useful in preventing fibrosis development; however, extreme dysregulation of the immune system towards either Th1 or Th2 has been shown to increase pathology and early mortality. Most importantly, maintaining a balance in Th2 effects is critical for preventing excessive pathology.

Understanding the mechanisms by which schistosomes dampen the immune response and elucidating their functions will aid in developing new strategies for the treatment and prophylaxis of fibrosis and other chronic inflammatory conditions.
